# Image classification optimization technology based on differentiable neural architecture search improvement model

**DOI:** 10.1371/journal.pone.0329480

**Published:** 2025-08-13

**Authors:** Yuxuan Ji, Wenshu Li, Nan Yu

**Affiliations:** College of Information Engineering, Shanghai Maritime University, Shanghai, China; Jiangsu Open University, CHINA

## Abstract

Image classification, as the core task of computer vision, has broad application value in fields such as medical diagnosis and intelligent transportation.However, the ability of differentiable neural architecture to search (NAS) for local information is weak, which limits the accuracy and long-distance information capture capability of the algorithm. Therefore, based on this, the study introduces visual attention mechanism and proposes an improved model that replaces the original convolution operator and adds residual structure in the macro structure to enhance the model’s information acquisition ability and classification accuracy. The research results show that after 600 rounds of training on the CIFAR-10 dataset, the final accuracy of the improved model reached 97.2%. The runtime memory usage on the CIFAR-100 dataset is only 44.52%, a decrease of 44.56% compared to the baseline model. In the testing on the ImageNet dataset, the classification accuracy of the research model is 94.01, the search parameter required is only 4.8MB, the search time is shortened to 0.5d, and the minimum number of floating-point operations is 3.7G, significantly better than other mainstream algorithms. The above results indicate that the research method can effectively solve the shortcomings of traditional differentiable neural architecture search in local and remote information acquisition capabilities, providing important technical support for improving the accuracy and efficiency of image classification technology.

## 1. Introduction

Image classification is one of the core tasks of computer vision, aimed at automatically identifying and annotating the main objects or scenes in images [1]. Image classification technology has important practical significance in today’s digital society, with a wide range of applications covering multiple key fields, greatly improving work efficiency and intelligence. For example, image classification technology can be used for medical image analysis to assist doctors in disease diagnosis. Additionally, in the realm of autonomous systems and intelligent transportation, this technology plays a crucial role in identifying key elements such as roads, vehicles, and pedestrians. Such capabilities enable intelligent decision-making processes, enhancing safety and efficiency in these sectors [2,3]. Convolutional Neural Network (CNN) plays a core role in image classification. By simulating the working mechanism of the human visual cortex, it can efficiently extract local features of images and combine them into global information, achieving high-precision classification tasks [4]. With the continuous expansion of application scenarios, many high-performance neural network architectures have emerged. However, traditional neural network architectures rely on manual design by experts, which requires significant time and effort, restricting the use of CNN [5]. To address this issue, numerous scholars have begun to explore automated design of neural network architectures, and neural network architecture search algorithms have emerged. The Differential Architecture Search (DARTS) algorithm stands out among many Neural Architecture Search (NAS) algorithms, as it utilizes gradient strategies to optimize architecture parameters, greatly reducing computational resource consumption [6]. However, the original convolution operator of DARTS has a weak ability to obtain local information, which leaves considerable space for optimization in the context of the accuracy of the algorithm and its ability to obtain long-distance information. Therefore, the study proposes an improved DARTS search model based on the Visual Attention Network (VAN) architecture, DARTS-VAN, to achieve efficient and accurate image classification. Based on the initial DARTS architecture, this model replaces the original operator with an operator that adds standard convolution. On this basis, residual structures are added to the macro structure to improve the model’s information acquisition ability and accuracy. This research endeavors to enhance the model’s information acquisition and transmission proficiencies by refining the neural network architecture search algorithm. The primary objective is to elevate the precision of search outcomes, subsequently propelling advancements in image classification technology.

The article is categorized into four sections. The first section introduces the current study on neural network architecture search worldwide and its application in image classification. The second section mainly introduces the principle and construction process of the DARTS-VAN proposed in the research. The third section conducts experiments on the capability of the DARTS-VAN model proposed in the study to verify its feasibility. The last section is a generalization and discussion of the article.

## 2. Related works

The neural network architecture search algorithm is an automated approach for finding the optimal neural network structure, which can automatically explore different combinations of network architectures through the algorithm to find the most suitable neural network model for specific tasks [7]. The NAS algorithm not only significantly improves the capability and efficacy of the model, reduces the time cost of manual design, but also optimizes the model under resource limited conditions, promoting the popularization and innovation of deep learning technology [8]. Researchers around the world have conducted extensive exploration on neural network architecture search algorithms, with fruitful results. Z. Ding et al. proposed a broad NAS method based on generalized CNNs to deal with the problems of long training time and performance degradation caused by deep scalable architectures in NAS. This method achieved higher search speed and better model performance by designing a shallower but broader network structure [9]. Y. Bian et al. proposed a new sub-architecture ensemble pruning method in NAS to address the issue of high computational costs. This method utilized diversity to implement sub-ensemble architectures in smaller sizes, thereby optimizing the efficiency and resource utilization of neural network design [10]. In response to the high computational cost of neural structure search, J. Dong et al. proposed an efficient meme algorithm for automatic CNN architecture search. The algorithm presented incorporates a unit-centric architecture search space alongside novel strategies for both comprehensive and targeted searches, effectively improving the efficiency and practicality of architecture search [11]. C. Wei et al. proposed an evolutionary algorithm guided by neural predictors to address the issues of high computational costs and insufficient exploration capabilities in NAS. This algorithm enhanced exploration capabilities and achieved efficient optimization of architecture search by designing graph-based uncertainty estimation networks and graph neural network predictors [12].

The NAS algorithm finds the optimal network structure in image classification through automated means. It utilizes search space, strategies, and evaluation mechanisms to automatically explore and optimize CNN architectures, reducing reliance on expert knowledge. This method significantly promotes the development of image classification technology, providing new avenues for achieving higher accuracy and efficiency. In response to the problems of deep learning models relying on pre-trained models, limited architecture design, and insufficient datasets in remote sensing image scene classification, C. Peng et al. proposed a novel architecture search structure for automatically designing CNN architectures. This method achieved efficient discovery of optimal network architectures in continuous search spaces through double-layer optimization and architecture regularization [13]. Q. Zhang proposed an improved ResNet34 model to solve the problem of traditional machine learning methods being difficult to recognize plant disease and pest images, especially when dealing with small sample sizes, blurry features, and numerous categories. The experimental results showed that the number of parameters in the model decreased by 85.37%, and the computational load decreased by 84.51% [14]. Q. Zhang et al. designed a progressive sample selection framework with contrast loss for noise labeling, called PSSCL, where the first stage identified a small clean dataset through a long-term confidence detection strategy, and the second stage aimed to improve performance by expanding this clean dataset. Compared with the most advanced methods, PSSCL showed significant improvements in various benchmark tests [15]. Q. Zhang et al. proposed a balanced partitioning and training framework called BPT-PLR, which combined pseudo label relaxation contrastive loss, to address the class imbalance in partitioned data subsets and the optimization conflict between unsupervised contrastive representation learning and supervised learning. Extensive experiments with state-of-the-art methods showed that BPT-PLR could achieve optimal or near optimal performance [16].

To sum up, researchers around the world have conducted many beneficial explorations on the NAS algorithm, and this algorithm is commonly applied in the area of image classification. However, although the differentiable NAS algorithm solves the high computational cost problem of traditional NAS algorithms, there is still a lot of room for optimization. This is due to the weak local information acquisition ability of its original convolution operator, which limits the accuracy and ability to obtain long-distance information of the algorithm. Therefore, the study proposes DARTS-VAN. On the basis of the DARTS architecture, this model uses a Large Kernel Attention (LKA) mechanism that does not include attention connections to improve the convolution operator, and introduces residual structures in the macro architecture to enhance the model’s feature acquisition ability and accuracy, which is innovative.

## 3. A differentiable NAS model based on VAN

In the process of building DARTS-VAN, two key aspects were explored in depth. Firstly, to address the problem of insufficient long-range information acquisition capability of the original DARTS, improvements were made based on the VAN architecture. Secondly, in the macro architecture, information transmission was optimized by adding residual structures.

### 3.1 Optimization of convolutional operators based on VAN in DARTS-VAN

**Fig 1:**
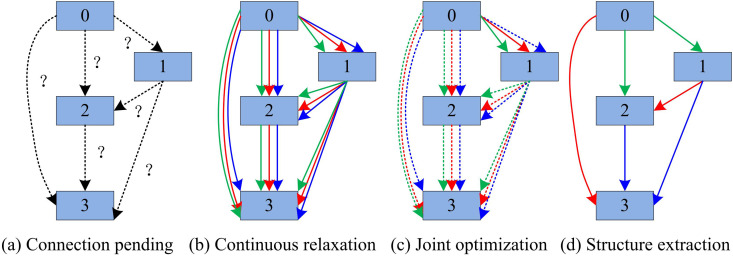
DARTS algorithm process.

The DARTS algorithm reduces the need for manual design through automated search, lowers design time and cost, and makes neural network design more efficient and scalable [17]. In image classification, this method can quickly find the optimal architecture suitable for a specific dataset, improving the practicality and adaptability of the model [18]. However, the original convolution operator of DARTS has a weak ability to obtain local information, which makes its ability to obtain long-distance information average and affects the accuracy of the algorithm [19]. To tackle this problem, research has improved the original operator through a VAN architecture based on an LKA mechanism. To elaborate on the improvement process of DARTS, the study first introduces the basic architecture of DARTS. DARTS employs a continuous relaxation technique for architecture representation, enabling efficient architecture exploration through gradient-based optimization. Rather than seeking a predefined set of discrete architecture candidates, the search space is transformed into a continuous domain, facilitating the optimization of architecture performance on the validation set via gradient descent [20,21]. The DARTS algorithm process is shown in Fig 1.

As shown in Fig 1, the DARTS algorithm includes four stages: connection pending, continuous relaxation, joint optimization, and structure extraction [22]. The edge operations in Fig 1(a) are initially unknown, and in the continuous relaxation stage, the search space is continuously relaxed by placing a mixture of candidate operations on each edge. Then, by tackling a two-layer optimization issue, the mixed chance and network weights are jointly optimized. Finally, the final structure is summarized from the learned mixed probabilities. DARTS progressively softens its initial discrete search approach, leveraging gradients to effectively refine the architecture search domain [23]. For the discrete search strategy, each intermediary node can be depicted as illustrated in equation (1).


$$x^{\left(j\right)}=\sum_{i<j}o^{\left(i,j\right)}(x^{\left(j\right)})$$ (1)

In equation (1), x represents a node in the directed graph, and DARTS follows a modular-based exploration domain for seeking two types of Cell structures. Each Cell is treated as a directed, non-cyclic graph consisting of N vertices: G=(V,E). 0<j<i<N−1 represents the presence of an action $o^{\left(i,j\right)}$ that takes the feature representation $x_{i}$ as input and outputs $x_{j}$. The DARTS algorithm softens the choice of potential operations into a softmax over all feasible operations, thereby making the discrete search strategy continuous. After the mixing operation ${\bar{\ell }}_{i,j}$, the operational feature map x can be described as:


$${\bar{\ell }}_{i,j}(x_{i})=\sum_{o\in \ell }\frac{exp({\alpha_{o}}^{\left(i,j\right)})}{\sum_{o^{\prime }\in \ell }exp(\alpha_{o^{\prime }}^{\left(i,j\right)})}o(xi)$$ (2)

**Fig 2:**
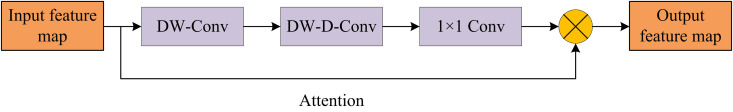
LKA model structure.

In equation (2), ${\alpha_{o}}^{\left(i,j\right)}$ represents the weight of the operation o on the directed edge in the directed acyclic graph. Therefore, neural network structure search is transformed into finding the optimal solution for a collection of uninterrupted variables. Despite the fact that the DARTS algorithm employs gradients to optimize architectural parameters for the purpose of searching network structures, significantly curtailing the consumption of computational resources, it exhibits a weakness in capturing local information. Consequently, this limitation results in an average performance when it comes to acquiring long-distance information [24]. This study improves DARTS based on the attention mechanism of LKA, replacing the original two operators with standard convolution operators. The LKA structure introduces attention mechanisms, especially LKA mechanisms, aimed at expanding the receptive field of the model to focus on a wider range of contextual information. Its model structure is shown in Fig 2.

As shown in Fig 2, the LKA structure consists of two parts: large kernel convolution kernel and self attention mechanism [25]. The convolution kernel includes three convolution operators, namely standard convolution DW-Conv, spatial long-distance convolution DW-D-Conv, and channel convolution 1 × 1Conv [26]. The structural design of LKA aims to expand the receptive range of neural networks, enabling them to capture a wider range of contextual information. The core idea of this mechanism is to enable each node to not only rely on local regions when processing information, but also consider a larger range of data points through special design [27]. The LKA mechanism enables each node to evaluate the importance of information from distant locations and adjust its own activation level based on these evaluations. To achieve this, the network calculates the correlation strength between different locations, emphasizing important areas while not ignoring information from other parts. Then, these weighted information are aggregated to generate richer and more comprehensive feature representations. This method helps the model better understand the overall layout and complex relationships of the image, while maintaining attention to details. In this way, the model can process multi-scale information more efficiently with limited resources, improving task performance. The formula representation of the LKA model is shown in equation (3).


$$\begin{array}{c} Attention=Conv1\times 1(DW-D-Conv(DW-Conv(F)))\\ Output=Attention⊗F \end{array}$$ (3)

**Fig 3:**
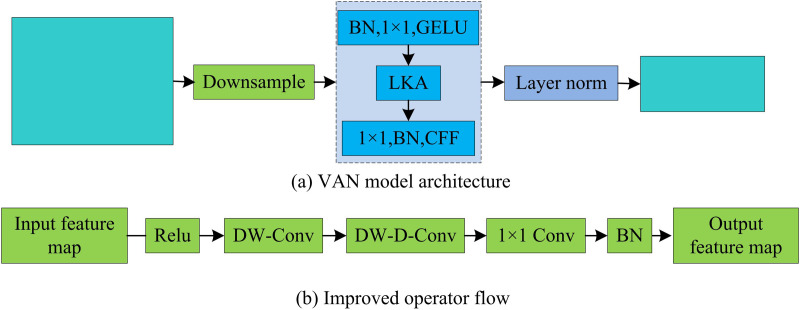
VAN model architecture and improved operator flow.

In equation (3), $F\in R^{C\times H\times W}$ represents the input feature, $Attention\in R^{C\times H\times W}$ denotes the attention feature, and ⊗ represents the dot product of the element. LKA not only has the good spatial perception and feature expression ability of convolutional networks, but also has the advantage of global dependency relationship of self attention mechanism, which expands the receptive field of images and shows good adaptability in both spatial and channel dimensions. The architecture of the VAN model based on LKA and the optimized internal operation flow of the operator are shown in Fig 3.

As shown in Fig 3(a), VAN consists of four parts, namely downsampling, batch normalization operation, attention mechanism, and re-batch normalization. This structure enables VAN to effectively capture important information in images and extract features at different resolutions. Fig 3(b) shows the internal operation flow of the operator optimized based on VAN. The input feature map initially undergoes the Rectified Linear Unit (ReLU) activation function, a prevalent choice in CNNs. If the input value falls below zero, the output is zero. Conversely, if the input value is zero or positive, the output matches the input value. Its expression is shown in equation (4).


$$y_{R}=\max(0,x_{R})$$ (4)

In equation (4), max(·) represents the comparison function that takes the maximum value. $x_{R}$ and $y_{R}$ respectively represent the input and output of the ReLU activation function, $y_{T}\in [0,+\infty )$. The ReLU function solves the gradient vanishing problem to some extent, while also alleviating the overfitting problem. Subsequently, a series of operations are carried out sequentially, including standard convolution, dilated convolution, pointwise convolution, and batch normalization. Ultimately, a fresh feature map is generated as the output. The process of improving the operator can be expressed as equation (5).


$$\left\{ \begin{array}{c} F\prime =Relu\left(F\right)\\ F^{\prime \prime }=Conv_{1\times 1}(DW-D-Conv(DW-Conv(F^{\prime })))\\ Output=BN(F^{\prime \prime }) \end{array}\right.$$ (5)

In equation (5), F means the input feature map, and BN denotes the batch normalization operation, which is generally used after the convolutional layer and before the activation layer. The calculation for the normalization adjustment is in equation (6).


$$\left\{ \begin{array}{c} \mu_{N}=\frac{1}{m}\sum_{i=1}^{m}x_{i}\\ \sigma_{N}^{2}=\frac{1}{m}\sum_{i=1}^{m}(x_{i}-\mu_{N}{)}^{2}\\ {\tilde{x}}_{i}=\frac{x_{i}-\mu_{N}}{\sqrt{\sigma_{N}^{2}+\varepsilon }} \end{array}\right.$$ (6)

In equation (6), $\mu_{N}$ and $\sigma_{N}^{2}$ respectively denote the mean and variance of a small batch input $N=\left\{x_{1},x_{2},...,x_{m}\right\}$, and ${\tilde{x}}_{i}$ is the input value after normalization adjustment. The second step of the BN approach is to introduce two learnable parameters γ and β, and then perform a linear transformation on the normalized input data, as shown in equation (7).


$${\tilde{y}}_{i}=\gamma {\tilde{x}}_{i}+\beta$$ (7)

In equation (7), ${\tilde{y}}_{i}$ represents the output of BN. When $\gamma^{2}=\sigma_{N}^{2}$, $\beta =\mu_{N}$, it is possible to achieve equivalent transformation while preserving the distribution information of the original input features.

### 3.2 Introduction of residual structure in DARTS-VAN differentiable NAS

**Fig 4:**
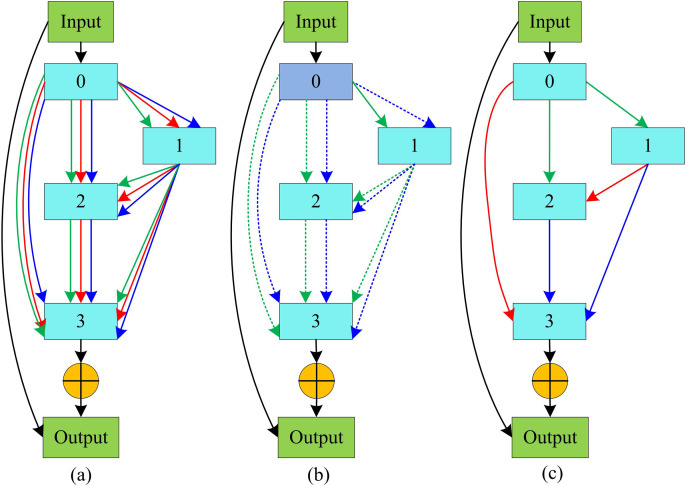
DARTS-VAN model unit search process.

After replacing the original operators of DARTS with a visual attention architecture based on LKA, the local information capability of the model has been enhanced. In the DARTS unit, due to the mixed operation performed between nodes, embedding the self attention mechanism into a single operator not only significantly increases the computational burden, but also may cause the problem of gradient explosion. Therefore, to further optimize the DARTS-VAN model, it is necessary to remove attention connections in LKA and add residual structures in the macro structure to improve the overall ability of the algorithm and achieve stronger information acquisition capabilities. The unit search process of the DARTS-VAN model with residual structure added is shown in Fig 4.

**Fig 5:**
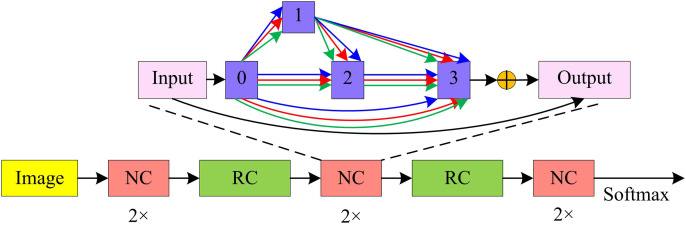
Schematic diagram of DARTS-VAN model structure.

As shown in Fig 4, after adding the residual structure, the internal structure of the cell remains in the shape of a directed, non-cyclic graph. Unlike DARTS, the input feature map generates a replica, which is accumulated with the node feature map and then outputs the result. Fig 4(a) shows the initialization phase of the search unit, during which all connections between nodes are refined into specific operators. Fig 4(b) reflects that during the search process, each operator will receive different weight assignments based on their importance. Fig 4(c) shows the situation after the search phase is done, where only the operator connection with the highest weight is retained between nodes. The overall framework of the DARTS-VAN model proposed in the study is shown in Fig 5.

As shown in Fig 5, the study introduces Reduction units at one-third and two-thirds of the nodes in the network model to increase the number of channels and reduce the size of the feature map. In the evaluation and validation phase, the Normal Cells and Reduction Cells explored by the model will be expanded and stacked in a predetermined order to construct a super network structure. The search space of DARTS-VAN is still based on unit stacking construction, where each unit is a directed acyclic graph containing 7 nodes representing potential representations of network layers, such as feature maps in the feature matrix. Each node is connected by an edge, and each edge is composed of a mixture of candidate operations. Specifically, the search space of DARTS-VAN includes many operators, with no operator indicating that no operation is performed between nodes, meaning there are no connected edges between nodes. The max pooling and average pooling operators use a 3 × 3 window for pooling. The depth-wise separable convolution operator is based on depth-wise separable convolutions using 3 × 3 and 5 × 5 convolution kernels. The large kernel convolution operation is implemented by standard convolution, dilated convolution, and point-wise convolution, with receptive fields equivalent to 5 × 5 and 9 × 9 convolution kernels, respectively. These operators constitute the search space of DARTS-VAN, and by optimizing the architecture parameters, the model can make choices among these operations to form the final network architecture.

**Fig 6:**
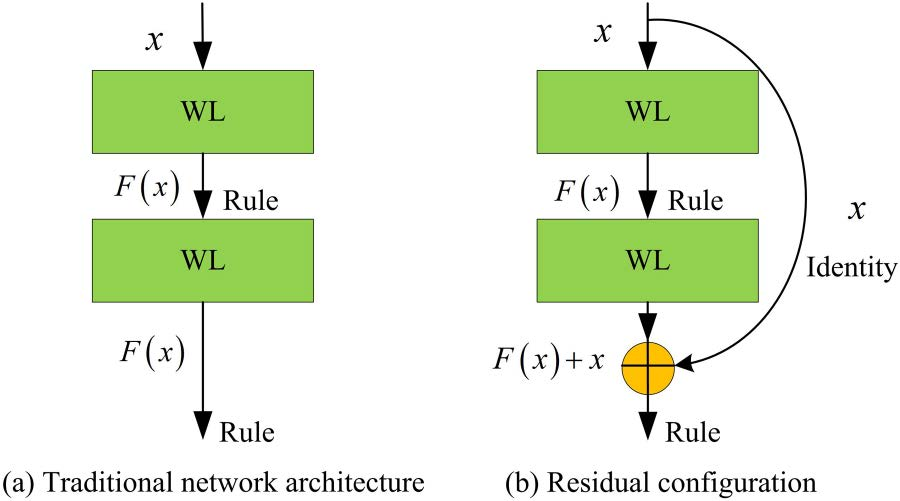
Residual configuration.

The DARTS-VAN model enhances the capability to extract local information in detail by using a large convolution kernel operator, while incorporating residual structures into the overall architecture, achieving more powerful communication of remote information. Residual Network (ResNet) is a deep learning model suggested by Kaiming He et al. [28]. The fundamental concept of ResNet revolves around addressing the issue of deterioration in the training of deep neural networks, specifically where the model’s performance may surprisingly diminish as the network’s layer count escalates [29]. ResNet performs well in multiple computer vision tasks, including visual categorization, object localization, and partitioning [30]. There are multiple variants of the ResNet family, such as ResNet-18, ResNet-34, ResNet-50, and ResNet-101, whose main differences lie in the complexity and breadth of the network. With the increase of network depth, models are able to learn more complex features, but meanwhile, they also require more computing resources [31]. The residual configuration is shown in Fig 6.

In Fig 6, unlike conventional network structures, residual structures introduce shortcut connections (also referred to as bypass connections or shortcut connections) to allow the output of a certain layer to be directly transmitted across layers to the input of subsequent layers. This ensures that even if some intermediate layers do not undergo significant feature transformations, the information from the previous layer can be transmitted without loss. This mechanism significantly reduces the risk of gradient vanishing. The mapping formula of the residual network is in equation (8).


H(x)=F(x)+x (8)

In equation (8), H(x) is the observed value, F(x) is the residual function, and x is the estimated value, which is the feature map of the residual output from the previous layer. The DARTS-VAN model with added residual structure exhibits higher stability and accuracy in neural network architecture search, mainly reflected in three aspects. Firstly, through skip connections, the residual structure directly transfers the information from the previous layer to the subsequent layers, effectively alleviating the problem of gradient vanishing and making it easier for gradients to propagate in the network. Secondly, in DARTS-VAN, the residual structure enhances the robustness of the search process by adding auxiliary skip connections and eliminating the advantages of skip connections, ensuring a more fair competition among all operations. Finally, residual frameworks empower the network to grasp more sophisticated and profound feature expressions, significantly improving network performance while accelerating model training.

## 4. Simulation experiment and analysis of search improvement model for differentiable neural architecture

To confirm the validity of the proposed DARTS model based on VAN, multiple databases were selected for training and simulation experiments, and the results were analyzed. The experimental platform selected a deep learning framework based on Pytorch, and the experimental equipment and related parameters were described in detail, as represented in Table 1.

**Table 1:** Experiment environment and parameter.

Experimental environment		
Configuration item		Configuration details
Processor		Interl Corei7-8750H
Graphics processing unit		GeForce RTX 3090 Ti(24GB)GPU
Internal memory		64G
Hard disk		500G
Operating system		Ubuntu18.04
Experimental parameter		
Search phase	Channel number	16
	Training rounds	50
	Batch size	64
	Learning rate	0.025
	Momentum parameter	0.9
	Weight decay rate	0.0003
Evaluation phase	Channel number	36
	Training rounds	600
	Batch size	128
	Learning rate	0.025
	Momentum parameter	0.9
	Weight decay rate	0.0003

**Fig 7:**
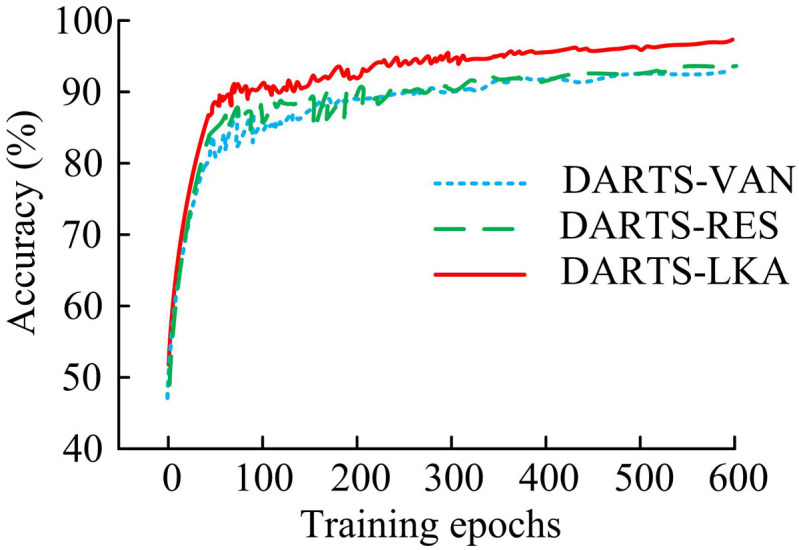
Accuracy test results.

According to Table 1, the hardware setup included an Intel Core i7-8750H processor, a GeForce RTX 3090 Ti Graphics Processing Unit (GPU) with 24GB of memory, 64GB of RAM, a 500GB hard drive, and Ubuntu 18.04 as the operating system. In the search stage, the model was configured with 16 channels, 50 training epochs, a batch size of 64, a learning rate of 0.025, a momentum of 0.9, and a weight decay of 0.0003. During the verification phase, the number of channels was increased to 36, with 600 training rounds, a batch size of 128, the same learning rate and momentum, and weight decay rate. The main goal of the search phase is to quickly explore and evaluate a large number of candidate architectures to determine the optimal network structure. At this stage, multiple architecture combinations need to be evaluated, and the computational cost is high. Therefore, the training epochs are limited to 50 epochs,. This enables the search process to iterate and adjust architecture parameters more quickly, thereby converging to a promising architecture more quickly. The goal of the search phase is to comprehensively verify the performance of the optimal architecture found during the search phase. In order to obtain more accurate performance evaluation, it is necessary to train the model for a longer period of time. Increasing the training epochs (600 epochs) can ensure that the model weights are fully optimized, thereby improving classification accuracy and stability. To ensure the reliability of the experimental results, and inter group differences were verified through paired t-tests. Initially, ablation experiments were conducted to assess the effectiveness of the enhanced modules and their influence on network performance. The baseline model’s convolution operator in DARTS-LKA was improved using the LKA mechanism, followed by the introduction of residual structure in DARTS-Res. From this, the ablation experiment results can be obtained, as shown in Fig 7, to test the effectiveness of different improved modules on the entire model.

As shown in Fig 7, after 600 rounds of training, the final accuracy of the DARTS-LKA model, which improved the convolution operator of DARTS based on the LKA algorithm, reached 93.5%. The final accuracy of the DARTS-Res model, which introduced residual structure, was 94.1%. The research model DARTS-VAN improved the convolution operator and added residual structure, resulting in a final accuracy of 97.2%. Overall, the research model could converge in a shorter period of time and had better overall stability. The final accuracy within a given training round was also significantly higher than the previous two. This is because DARTS-LKA improves the model’s ability to obtain local information by introducing the LKA mechanism, but relying solely on the LKA mechanism is still insufficient to fully utilize the model’s performance. After introducing residual structures, DARTS-VAN effectively alleviates the gradient vanishing problem by allowing information to be directly transmitted across multiple layers, enabling the model to better train deep networks and further improve performance. DARTS Res only introduces residual structures, and due to the lack of improved convolution operators, its ability to obtain local information is insufficient. The study tested the precision (P), recall (R), F1 score (F1), and overall accuracy (OA) of the three models mentioned above, as well as the baseline model DARTS. The CIFAR-10 and CIFAR-100 datasets were chosen as the test datasets. The results are in Table 2.

**Table 2:** Test results on different datasets.

The results of different models on the CIFAR-10 dataset				
Approaches	P	R	F1	OA
DARTS	0.889	0.876	0.890	0.974
DARTS-LKA	0.925	0.881	0.903	0.988
DARTS-Res	0.930	0.884	0.906	0.992
**DARTS-VAN**	0.935	0.887	0.912	0.994
The results of different models on the CIFAR-100 dataset				
Approaches	P	R	F1	OA
DARTS	0.873	0.799	0.841	0.969
DARTS-LKA	0.908	0.868	0.886	0.973
DARTS-Res	0.912	0.872	0.895	0.976
**DARTS-VAN**	0.926	0.880	0.905	0.980

**Fig 8:**
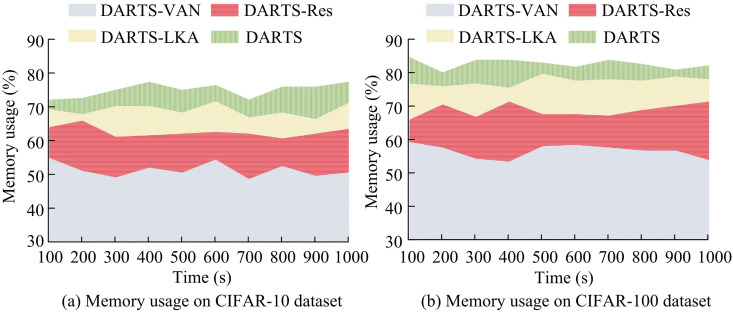
Comparison result of memory usage.

In Table 2, on the CIFAR-10 dataset, the P, R, F1, and OA of the baseline model DARTS were 0.889, 0.876, 0.890, and 0.974. The P, R, F1, and OA of the DARTS-LKA model with improved convolution operator were 0.925, 0.881, 0.903, and 0.988. The P, R, F1, and OA of the DARTS-Res model with introduced residual structure were 0.930, 0.884, 0.906, and 0.992. The P, R, F1, and OA of the DARTS-VAN model with improved convolution operator and added residual structure were 0.935, 0.887, 0.912, and 0.994, which were superior to other models. On the CIFAR-100 dataset, the P, R, F1, and OA of the research model were 0.926, 0.880, 0.905, and 0.980. Compared with the baseline model, the four detection indicators improved by 6.1%, 10.1%, 7.6%, and 1.1%. Furthermore, to verify the operational efficiency of the improved research model, the memory usage of different models during runtime was tested on the CIFAR-10 and CIFAR-100 datasets, and the outcomes are in Fig 8.

**Fig 9:**
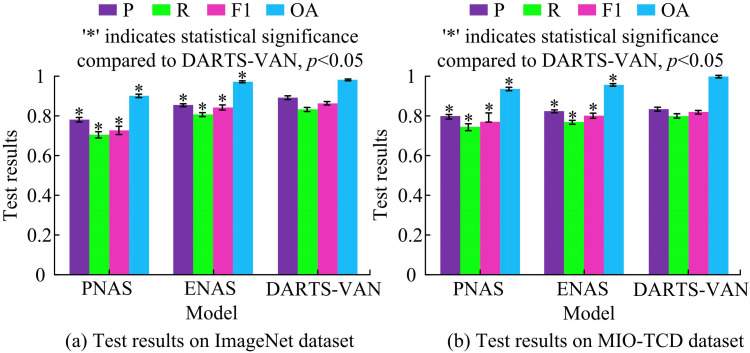
Test results for P, R, F1, and OA.

As shown in Fig 8, on the CIFAR-10 dataset, the average memory usage of the baseline model DARTS was 73.48%, the average memory usage of the DARTS-LKA model was 67.23%, the average memory usage of the DARTS-Res model was 57.44%, and the average memory usage of the research model was 41.89%, a decrease of 42.99% compared to the baseline model. On the CIFAR-100 dataset, the average memory usage of the research model was 44.52%, a decrease of 44.56% compared to the baseline model. The above results are generated because the research method introduces LKA mechanism and residual structure, which reduces redundant calculations and parameters while maintaining high performance, making the model more suitable for running in resource constrained environments. A comparative experiment was conducted to test the generalization ability of different models on the ImageNet dataset and MIO-TCD dataset. The test indicators were P, R, F1, and OA. The comparison methods selected were Efficient NAS (ENAS) and Progressive NAS (PNAS). The results are shown in Fig 9.

According to Fig 9(a), on the ImageNet dataset, the P, R, F1, and OA of the PANS model were 0.771, 0.725, 0.748, and 0.904. The P, R, F1, and OA of the ENAS model were 0.849, 0.815, 0.838, and 0.972. The P, R, F1, and OA of the study model were 0.892, 0.836, 0.857, and 0.981. According to Fig 9(b), on the MIO-TCD dataset, the P, R, F1, and OA of the proposed DARTS-VAN model were 0.832, 0.804, 0.825, and 0.991, which were superior to other models. In addition, from a statistical significance perspective, in both datasets, the indicators of the research method showed significant statistical significance (*p* < 0.05) compared to other mainstream methods. This was because the research method introduced VAN and residual structure, which enhanced the model’s ability to extract image features and adapt to complex data, thereby improving classification performance. Overall, the test results of the research model on the ImageNet dataset and MIO-TCD dataset were better than the comparison model, demonstrating good generalization ability. To comprehensively evaluate the performance of the research method, Normalization Mean Error (NME) and Failure Rate (FR) were introduced for evaluation, and complex and challenging datasets such as ImageNet, MIO-TCD, CIFAR-10, CIFAR-100, and Tiny ImageNet were used for testing. NME is an indicator used to evaluate the performance of a model by calculating the normalized error between the predicted results and the true labels, as shown in equation (9).


$$NME=\frac{1}{N}\sum_{i=1}^{N}\frac{∥y_{i}-{\hat{y}}_{i}∥}{∥y_{i}∥+\varepsilon }$$ (9)

**Fig 10:**
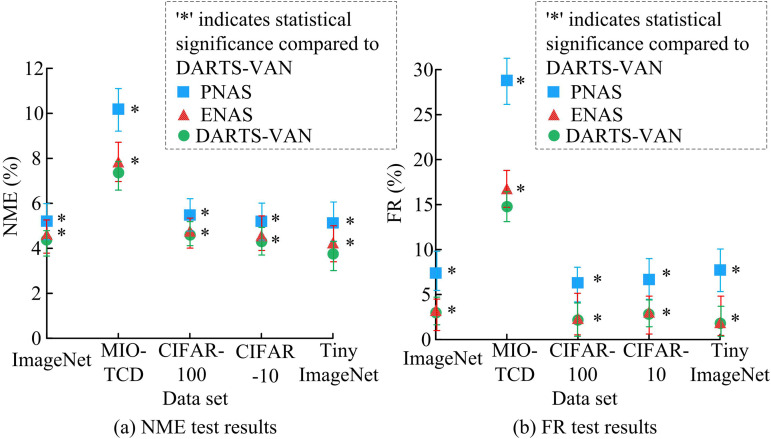
Test results for NME and FR.

In equation (9), $y_{i}$ and ${\hat{y}}_{i}$ respectively represent the true value and the model predicted value, ε representing the minimum constant. FR is the proportion of failed samples to the total sample size, used to measure the robustness of the model to difficult samples. The study also used ImageNet dataset, MIO-TCD dataset, CIFAR-10 dataset, CIFAR-100 dataset, and Tiny ImageNet dataset for testing, and the results are shown in Fig 10.

**Fig 11:**
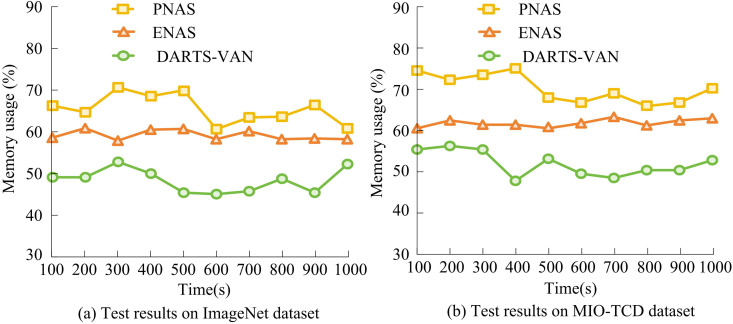
Comparison result of memory usage.

According to Fig 10, the NME and average FR of the research model on the five test datasets were 4.86% and 5.06%, respectively, which were lower than other algorithms. The MIO-TCD dataset is a vehicle classification dataset collected by thousands of traffic cameras deployed in Canada and the United States, representing typical visual data captured in urban traffic scenes today. The data is large and complex, resulting in high error and FRs on this dataset. However, overall, the DARTS-VAN model proposed in the study still had significantly lower error and FRs than other models, demonstrating superior performance. From statistical significance analysis, it can be seen that in different datasets, the NME and FR of DARTS-VAN are significantly lower than those of PANS and ENAS (*p* < 0.05). This is because in DARTS-VAN, LKA combines large kernel convolution with channel attention, allowing the model to capture both local details and global contextual information simultaneously, solving the problem of limited receptive field in traditional DARTS convolution operators. And residual structure optimization allows gradients to propagate directly across layers, solving the problem of gradient degradation in deep network training and making the search process more stable. The study tested the memory usage of different models during runtime on the ImageNet dataset and MIO-TCD dataset, and the results are shown in Fig 11.

According to Fig 11, after 1000 seconds of testing, the average memory usage of the research model on the ImageNet dataset was 48.81%, the average memory usage of the ENAS algorithm was 59.25%, and the average memory usage of the PNAS algorithm was 66.49%. On the MIO-TCD dataset, the average memory usage of the research model was 53.08%, the average memory usage of the ENAS algorithm was 62.74%, and the average memory usage of the PNAS algorithm was 70.33%. This is because the DARTS-VAN model adopts a gradient based differentiable architecture search method, which can find the optimal network architecture in a shorter time. This efficient search strategy not only reduces search time, but also optimizes the structure of the model, enabling it to utilize memory resources more efficiently during runtime. Finally, multiple mainstream NAS algorithms were selected for testing on the ImageNet dataset, with metrics including accuracy, memory parameter count, search evaluation time (GPU days), and Floating point operations (FLOPs).

Table 3 shows that the accuracy of DARTS-VAN reaches 94.01%, the search parameters only require 4.8MB, the search time is only 0.5 days, and the FLOPs value is 3.7G. This indicates that its computational resource consumption is very low when searching for network architecture, and it can quickly find the optimal network architecture, greatly improving search efficiency and reducing time and computational costs. At the same time, it requires less computation when processing data, which can improve the efficiency of the model while ensuring high accuracy, reduce hardware resource requirements, and make it more suitable for deployment and operation in practical applications. The Inception-v1 model has an accuracy rate of 83.52% and a relatively small parameter size of 7.9MB, which gives it an advantage in hardware resource limited environments and enables faster training and inference; Therefore, there is significant room for improvement in the accuracy of image classification. The MobileNet model is a lightweight neural network model with an accuracy of 83.07% and a parameter size of only 5.8MB, making it highly suitable for running in resource constrained environments such as mobile devices. However, its search efficiency and computational complexity are not as good as DARTS-VAN, so its performance may be limited in image classification tasks that require high accuracy. NASNet is a reinforcement learning based NAS model with an accuracy of 90.84%. Its search cost is very high, requiring 2050 GPU days, which makes its scalability poor in practical applications and difficult to find the optimal network architecture in a short period of time. The ENAS model has reduced search costs, but its search time is still relatively long, requiring 2000 GPU days, which still has certain limitations in practical applications. In addition, the FLOPs value of ENAS is 4.1G, which is lower than models such as NASNet. However, compared to DARTS-VAN, its computational complexity is still higher, which may result in the model requiring more computing resources at runtime, thereby affecting its efficiency and performance. The AmoebaNet model has a high accuracy of 91.50%, but its search cost is very high, requiring 3050 GPU days, making it difficult to find the optimal network architecture in a short period of time. The DARTS model has an accuracy of 91.28% and can quickly search for network architectures. Its search cost is relatively low, requiring only 4 GPU days, which makes it highly scalable in practical applications. But its computational complexity is still high, which may limit its efficiency and performance in practical applications. The accuracy of both PC-DARTS and P-DARTS models is very high, exceeding 92%, and the search cost is relatively low, but the corresponding computational complexity is still relatively high.

**Table 3:** Performance comparison of DARTS-VAN and mainstream NAS algorithms on ImageNet.

Model	Accuracy/%	Params(MB)	Search cost (GPU days)	FLOPs (G)	Type
Inception-v1	83.52	7.9	/	1.5	Manual
MobileNet	83.07	5.8	/	0.6	Manual
NASNet	90.84	5.9	2050	5.3	RL-based
ENAS	91.05	4.9	2000	4.1	RL-based
AmoebaNet	91.50	5.4	3050	7.2	EA-based
DARTS(second order)	91.28	4.6	4	4.8	GD-based
PC-DARTS	92.74	4.9	0.4	3.9	GD-based
P-DARTS	92.56	5.2	2.9	4.3	GD-based
**DARTS-VAN**	94.01	4.8	0.5	3.7	GD-based

## 5. Discussion and conclusion

A search model called DARTS-VAN, which introduced visual attention mechanism, was proposed to handle the poor local information acquisition ability of the original convolution operator in DARTS. Based on the initial DARTS architecture, this model replaced the original operator with an operator that added standard convolution, and added residual structure in the macro structure, thereby enhancing the model’s information acquisition ability and accuracy. Finally, the effectiveness and superiority of the above content were verified through experiments. The accuracy detection results on the CIFAR-10 dataset showed that after 600 rounds of training, the final accuracy of the improved research model reached 97.2%, which could converge in a short time and had better overall stability. The runtime memory usage on the CIFAR-100 dataset showed that the average memory usage of the research model was 44.52%, a decrease of 44.56% compared to the baseline model. The improved DARTS-VAN model had the lowest memory usage, which was beneficial for improving the computational efficiency of the model. The generalization ability of different models was tested on the ImageNet dataset and MIO-TCD dataset. The results showed that on the ImageNet dataset, the P, R, F1, and OA of the studied models were 0.892, 0.836, 0.857, and 0.981. On the MIO-TCD dataset, the P, R, F1, and OA of the proposed DARTS-VAN model were 0.832, 0.804, 0.825, and 0.991, which were superior to other models. Finally, multiple mainstream NAS algorithms were selected for testing on the ImageNet dataset. The outcomes indicated that the accuracy of the research model was 94.01%, the required parameters for search were 4.8MB, and the search time was only 0.5 days. In summary, the DARTS-VAN model proposed in the study could perform high-precision search on network architecture, had strong information acquisition ability, short search time, and superior performance. However, the search object of NAS was mainly CNN architecture, and there are many types of neural networks. In the future, it can be further expanded to other network structures, such as Recurrent Neural Network (RNN) and Generative Adversarial Network (GAN), which can be further studied.

## Supporting information

S1 File Minimal data set definition. (DOCX)
