# Supplementary material for: Image classification optimization technology based on differentiable neural architecture search improvement model
Source: PLoS One. 2025 Aug 13;20(8):e0329480. doi: 10.1371/journal.pone.0329480 (PMC12349122; doi:10.1371/journal.pone.0329480)
Supplement: S1 File — (DOCX) [file pone.0329480.s001.docx]

**The data in Figure 7**

| Epoch | Model | | |
| --- | --- | --- | --- |
|  | DARTS-LKA | DARTS-Res | DARTS-VAN |
| 0 | 47.6 | 48.5 | 52.7 |
| 100 | 58.7 | 62.3 | 68.9 |
| 200 | 78.4 | 81.6 | 86.2 |
| 300 | 86.9 | 88.5 | 92.7 |
| 400 | 90.3 | 91.8 | 95.4 |
| 500 | 92.1 | 93.5 | 96.6 |
| 600 | 93.5 | 94.1 | 97.2 |

**The data in Figure 8**

| Data set | Time (s) | DARTS | DARTS-LKA | DARTS-Res | DARTS-VAN |
| --- | --- | --- | --- | --- | --- |
| CIFAR-10 | 100 | 72.45 | 65.34 | 63.14 | 55.56 |
|  | 200 | 73.78 | 67.68 | 67.87 | 53.78 |
|  | 300 | 78.91 | 66.92 | 63.59 | 51.23 |
|  | 400 | 77.04 | 67.16 | 63.32 | 52.67 |
|  | 500 | 76.27 | 68.49 | 64.05 | 51.12 |
|  | 600 | 74.54 | 67.82 | 64.78 | 54.56 |
|  | 700 | 72.73 | 66.15 | 63.51 | 51.05 |
|  | 800 | 74.96 | 67.48 | 63.24 | 52.44 |
|  | 900 | 76.19 | 68.14 | 64.97 | 51.88 |
|  | 1000 | 78.42 | 73.81 | 65.72 | 50.32 |
| CIFAR-100 | 100 | 84.45 | 77.34 | 67.89 | 59.67 |
|  | 200 | 81.78 | 76.68 | 72.62 | 58.89 |
|  | 300 | 83.91 | 77.92 | 69.35 | 58.12 |
|  | 400 | 82.04 | 78.16 | 68.08 | 57.35 |
|  | 500 | 81.27 | 80.49 | 69.81 | 58.58 |
|  | 600 | 82.51 | 79.82 | 68..54 | 57.81 |
|  | 700 | 81.73 | 78.15 | 68.27 | 57.04 |
|  | 800 | 80.96 | 79.48 | 68.94 | 57.27 |
|  | 900 | 81.19 | 79.81 | 69.73 | 55.57 |
|  | 1000 | 82.42 | 78.14 | 71.46 | 53.73 |

**The data in Figure 9**

| Data set | Evaluation | PNAS | ENAS | DARTS-VAN |
| --- | --- | --- | --- | --- |
| ImageNet | P | 0.771±0.021 | 0.849±0.018 | 0.892±0.012 |
|  | R | 0.725±0.024 | 0.814±0.019 | 0.836±0.015 |
|  | F1 | 0.748±0.019 | 0.837±0.016 | 0.859±0.011 |
|  | OA | 0.904±0.014 | 0.972±0.008 | 0.981±0.005 |
| MIO-TCD | P | 0.802±0.023 | 0.818±0.019 | 0.832±0.013 |
|  | R | 0.788±0.024 | 0.801±0.021 | 0.804±0.014 |
|  | F1 | 0.791±0.022 | 0.812±0.018 | 0.826±0.012 |
|  | OA | 0.983±0.006 | 0.988±0.003 | 0.991±0.002 |

**The data in Figure 10**

| Test result | Data set | PNAS | ENAS | DARTS-VAN |
| --- | --- | --- | --- | --- |
| NME | ImageNet | 5.12 | 4.56 | 4.27 |
|  | MIO-TCD | 10.23 | 7.89 | 7.54 |
|  | CIFAR-10 | 5.77 | 4.53 | 4.51 |
|  | CIFAR-100 | 5.54 | 4.47 | 4.35 |
|  | Tiny ImageNet | 5.65 | 4.38 | 3.98 |
| FR | ImageNet | 7.89 | 3.45 | 3.12 |
|  | MIO-TCD | 28.45 | 16.78 | 14.56 |
|  | CIFAR-10 | 6.23 | 3.89 | 3.47 |
|  | CIFAR-100 | 6.78 | 3.45 | 3.34 |
|  | Tiny ImageNet | 8.45 | 3.67 | 3.45 |

**The data in Figure 11**

| Data set | Time (s) | PNAS | ENAS | DARTS-VAN |
| --- | --- | --- | --- | --- |
| ImageNet | 100 | 66.23 | 59.87 | 49.32 |
|  | 200 | 65.56 | 60.12 | 50.45 |
|  | 300 | 72.89 | 59.05 | 54.67 |
|  | 400 | 69.12 | 60.37 | 51.89 |
|  | 500 | 70.45 | 59.62 | 47.34 |
|  | 600 | 62.01 | 58.45 | 46.56 |
|  | 700 | 64.34 | 60.08 | 49.78 |
|  | 800 | 65.07 | 58.76 | 51.65 |
|  | 900 | 65.87 | 59.23 | 48.12 |
|  | 1000 | 61.54 | 57.09 | 54.23 |
| MIO-TCD | 100 | 76.23 | 61.45 | 56.87 |
|  | 200 | 75.67 | 63.12 | 57.24 |
|  | 300 | 76.11 | 62.79 | 55.31 |
|  | 400 | 77.89 | 63.02 | 47.45 |
|  | 500 | 68.45 | 62.58 | 53.95 |
|  | 600 | 66.01 | 62.14 | 53.65 |
|  | 700 | 67.56 | 63.67 | 52.78 |
|  | 800 | 66.02 | 62.34 | 53.89 |
|  | 900 | 68.45 | 62.01 | 53.23 |
|  | 1000 | 70.89 | 63.98 | 54.36 |
